# Impact of Drinking Water Fluoride on Human Thyroid Hormones: A Case- Control Study

**DOI:** 10.1038/s41598-018-20696-4

**Published:** 2018-02-08

**Authors:** Zohreh Kheradpisheh, Masoud Mirzaei, Amir Hossein Mahvi, Mehdi Mokhtari, Reyhane Azizi, Hossein Fallahzadeh, Mohammad Hassan Ehrampoush

**Affiliations:** 1 Environmental Science and Technology Research Center, Department of Environmental Health Engineering, Shahid Sadoughi University of Medical Sciences, Yazd, Iran; 2 Yazd Cardiovascular Research Center, Shahid Sadoughi University of Medical Sciences, Yazd, Iran; 3 School of Public Health, Tehran University of Medical Sciences, Tehran, Iran; 4 Center for Solid Waste Research, Institute for Environmental Research, Tehran University of Medical Sciences, Tehran, Iran; 5 Department of Endocrinology Shahid Sadoughi University of Medical Sciences, Yazd, Iran; 6 Department of Biostatistics, Shahid Sadoughi University of Medical Sciences, Yazd, Iran

## Abstract

The elevated fluoride from drinking water impacts on T_3_, T_4_ and TSH hormones. The aim was study impacts of drinking water fluoride on T_3_, T_4_ and TSH hormones inYGA (Yazd Greater Area). In this case- control study 198 cases and 213 controls were selected. Fluoride was determined by the SPADNS Colorimetric Method. T_3_, T_4_ and TSH hormones tested in the Yazd central laboratory by RIA (Radio Immuno Assay) method. The average amount of TSH and T_3_ hormones based on the levels of fluoride in two concentration levels 0–0.29 and 0.3–0.5 (mg/L) was statistically significant (P = 0.001 for controls and P = 0.001 for cases). In multivariate regression logistic analysis, independent variable associated with Hypothyroidism were: gender (odds ratio: 2.5, CI 95%: 1.6–3.9), family history of thyroid disease (odds ratio: 2.7, CI 95%: 1.6–4.6), exercise (odds ratio: 5.34, CI 95%: 3.2–9), Diabetes (odds ratio: 3.7, CI 95%: 1.7–8), Hypertension (odds ratio: 3.2, CI 95%: 1.3–8.2), water consumption (odds ratio: 4, CI 95%: 1.2–14). It was found that fluoride has impacts on TSH, T_3_ hormones even in the standard concentration of less than 0.5 mg/L. Application of standard household water purification devices was recommended for hypothyroidism.

## Introduction

Over the past decade, several studies have focused on the effects of environmental toxins on the human endocrine system, including the impact of fluoride on the thyroid gland^1,2^. Globally, millions of people suffer from thyroid-related problems. When the thyroid gland does not function properly, it can affect multiple aspects of our health^3–5^. The most important effect is thyroid complications in pregnant women. Uncontrolled hypothyroidism can raise the blood pressure during late pregnancy, increase the risk of miscarriage and preterm delivery, and affect brain development and growth rate^3–5^. The incidence of thyroid cancer has risen from 2% to 5% per decade. If this trend continues, thyroid cancer may become the fourth most common cancer in the United States by 2030^6^. The incidence rate of this type of cancer is two times more in high-income and middle-income countries as compared to low-income countries. It has a three to four time’s higher probability of occurring in women than in men^6^. The adverse effects of fluoride on animal and human health are well documented in the literature^7,8^. The effects of high fluoride ingestion through drinking water, green tea, and ambient air pollution on thyroid hormones (T3, T4, TH, and TSH) were investigated in both humans and animals. Some studies reported a reduction in the T4 and T3 levels as well as an abnormal increase in the TSH levels^9–15^. Ruiz-Payan *et al*.^16^ said that even at 1 mg/L of fluoride in water, T3 levels reduced in teenagers living in Northern Mexico^16^. During the years 2007–2016, the Parents of Fluoride Poisoned Children (PFPC) has reported over 190 studies on the effects of fluoride on thyroid hormones, including studies on both animals and humans^17^. Some studies have discovered the relation between dental fluorosis and thyroid disease^18–23^. The effect of thyroid hormones on learning memory was investigated in rats by Basha *et al*.^24^. They found that fluoride reduces the T4 and T3 levels, and has generational and cumulative effects on the development of the offspring^24^.

Yazd Greater Area (YGA) is located in the Yazd province of Iran, which uses groundwater as the primary water source. There are several wells in this region with different fluoride concentrations^25^. The halogen fluoride may enter the drinking water through human resources but its main medium is through natural resources, such as minerals, as well as geothermal and atmospheric means. The earth’s crust contains 0.3 g/kg of fluoride while the atmosphere has about 3 ng/m^3^ ^8,26,27^. The concentration of fluoride in drinking water is very important for the health of the people as it is one of the important resources containing this halogen^9^. Iran’s drinking water standard for fluoride is less than 1.5 mg/L in two liters per day for adults. This guideline has been recommended by the World Health Organization (WHO)^8^. This problem persists in the Third World and even in developed countries due to the lack of proper information about the potential effects of fluoride on human health. There is no published research in this field in Iran. The optimal dose for fluoride intake from drinking water depends on the various types of diets in different countries; it is also dependent on climate change. Meanwhile, thyroid diseases are related to human race and gender^28,29^. Therefore, it is beneficial to conduct this study in Iran. The purpose of this case-control study was to determine the correlation between thyroid hormones and the presence of fluoride in drinking water in YGA. This area was chosen because the wells in this area have water with different fluoride concentrations. Hence, the selection of cases and controls from the same region with similar diets, race, and gender, as well as climatic and geographical change, is possible.

## Results and Discussion

The spline GIS model was used to evaluate the distribution of fluoride levels in the drinking water samples from YGA. As mentioned, all the samples of drinking water from YGA had fluoride levels lower than the maximum permissible level of the world standard concentration and equal to the Iranian standard (0.5–1.5 mg/L)^8^.

**Figure 1:**
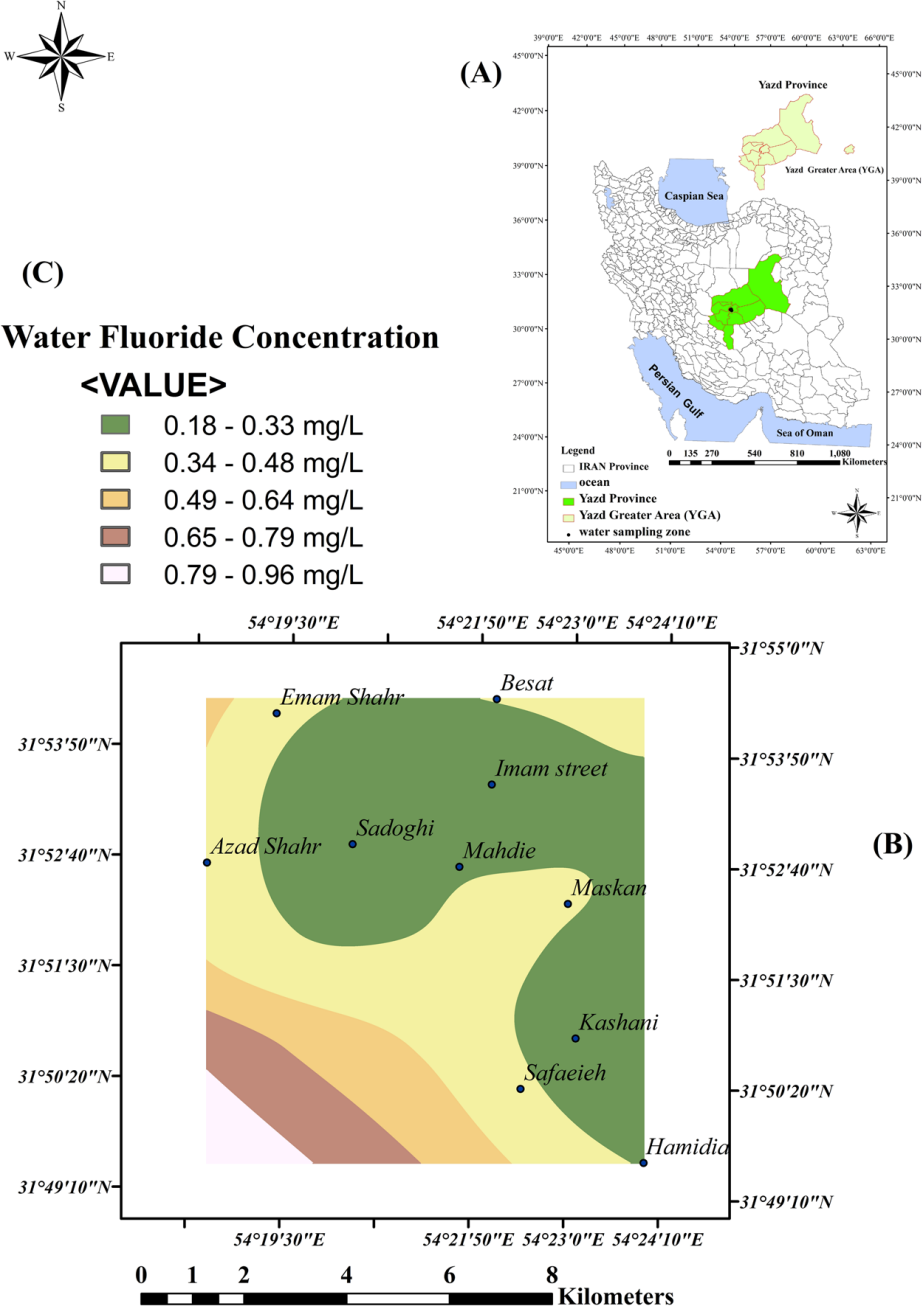
Zoning of fluoride levels in drinking water sampling in Yazd Greater Area (YGA) by Spline GIS model by Arc GIS 10 in Iran in 2016 (**A**) map of Iran and location of YGA (**B**) Spline GIS model for drinking water fluoride in ten distinct (**C**) water fluoride concentration.

The results are shown in Fig. 1. This figure shows the zoning of water samples and fluoride levels in different parts of YGA. As mentioned, 10 distinct locations were chosen according to the difference in the concentration of fluoride in drinking water. In each season, 30 samples were taken from 10 districts (three samples from each district). Hence, in two seasons, a total of 60 samples were collected (summer and winter). Each sample was tested thrice. The total number of tests to determine the fluoride concentration was 180. The Kolmogorov–Smirnov test was used to evaluate the normality of the data, and it was found that the data was not normally distributed for P < 0.05. Therefore, non-parametric tests (Kruskal–Wallis (were used to analyze the same. The median (interquartile range) of fluoride, and the temperature and pH of the drinking water have been shown in Table 1.

**Table 1:** Drinking water quality parameters of YGA in case and control groups, YGA (Yazd Greater Area), 2017.

**Variable**	**Case Median(IR)**	Control Median(IR)*	**P Value**
Fluoride	0.07 ± 0.38	0.07 ± 0.35	0.94
Temperature	0.6 ± 19.3	6 ± 19.3	0.16
pH	0.63 ± 7.3	0.56 ± 7.2	0.24

^*^IR, Interquartile range.

Out of the 8,724 YaHS samples that were studied until October 2015, a total of 693 (8%) participants had thyroid problems, while 70% had hypothyroidism. Among the participants who had no thyroid problems, as diagnosed by a doctor, 228 people were chosen and their TSH, T_3_, and T_4_ hormone levels were tested. The results showed that 213 of the participants were healthy, whereas 15 (6.6%) were sick. Among the latter, 11 (4.8%) suffered from hypothyroidism while four (1.8%) had hyperthyroidism. These 15 sick participants were removed from the controls. The distribution of different kinds of thyroid diseases in the 265 cases include 198 (74.7%) with hypothyroidism, 10 (3.7%) with hyperthyroidism, 27 (10.2%) with thyroid nodules, and one (0.37%) with thyroid cancer. As per the scope of this study, 198 (74.7%) participants with hypothyroidism were selected for the cases. The distribution frequency of hypothyroidism obtained by the chi-square test was statistically significant for the cases (P = 0.032) and controls (P = 0.024) at different participant locations in YGA.

Ten distinct locations were chosen according to the difference in the concentration of fluoride in drinking water. These ten distinct locations were contain: Sadoughi, Imam street, Kashani Street, Mahdieh Street, Hamidia Street, Be’sat Street, maskan Street, safaeieh Street, imam Shahr Street, Azad Shahr Street. The frequency distribution of fluoride was statistically significant for the cases (P = 0.001) and controls (P = 0.001). The frequency distribution of hypothyroidism based on the different levels of fluoride in drinking water was not significant for the cases (P = 0.13) and controls (P = 0.21) in YGA. The average amount of TSH and T3 hormones based on the fluoride levels in the range of 0–0.29 mg/L and 0.3–0.5 mg/L was significant. However, it was not significant for the T4 hormone in the case and control groups as shown in Table 2.

**Table 2:** The amount of T_4_, T_3_, and TSH hormones based on two levels of fluoride in drinking water in cases and controls, YGA (Yazd Greater Area), 2017.

**Variable**	Case median(IR)*		**P Value**	Control median(IR)*		**P Value**
	0–0.29 mg/L	0.3–0.5 mg/L		0–0.29 mg/L	0.3–0.5 mg/L	
T_4_	6.56 ± 2.2	7.6 ± 4.3	0.17	8.5 ± 1.2	8.6 ± 1.2	0.45
T_3_	115.3 ± 22	117.8 ± 36.6	0.19	135 ± 18.4	138.5 ± 21.6	0.026
TSH	11.85 ± 7	20.5 ± 12.8	0.003	2.2 ± 0.95	2.8 ± 0.9	0.001

^*^IR, Interquartile range. The normal range for T_3_ hormone is (78–180 ng/dL). The normal range for T_4_ hormone is (5.5–12.5 μg/dL). The normal range for TSH hormone is (0.17–4.5 mlU/L).

As shown in Table 2, the median ± interquartile range (IR) of TSH and T3 was significant on two levels of fluoride in drinking water (P < 0.05). Hence, it can be concluded that the halogen has an impact on human thyroid hormones. At a concentration of below 0.5 mg/L, however, it is not an important factor for hypothyroidism in YGA. This finding confirms the results of other studies^10–16,30^. The mean of the TSH hormone level, according to different study variables, is demonstrated in Table 3. For each of the questioned parameters of cases and controls, the OR, confidence interval (CI 95%), and p-value were examined across different case and control groups. The ones with a p-value less than 0.2 were used on the final logistic model. Finally, 14 parameters were entered into the final logistic model: sex, family history of thyroid disease, education and job status, quantity of drinking water, exercise, tobacco use, living place, and disease history, such as hyperlipidemia, diabetes, hypertension, polycystic, psychiatric, and depression. The adjusted odds ratio (OR), confidence interval (CI 95%), and p-value from the logistic model among the case and control groups were estimated for other fluoride intake sources, apart from water—such as toothpaste, mouthwashes, and some foods that contain fluoride (tea, cabbage, broccoli, turnip, soya, peanut, spinach, type of consumed fish, amount of consumed fish, type of consumed salt). Except for the 14 parameters that have been mentioned, other parameters had a p-value of more than 0.2, and were not used in the final logistic model. The final model was developed using multiple logistic regression modeling, as well as enter and forward LR methods. The results of the multiple logistic regression model with the p-value, adjusted OR, and confidence intervals (CI 95%) are shown in Table 4.

**Table 3:** Mean of TSH hormone according to different study variables, YGA (Yazd Greater Area), 2017.

**Characteristics**	**Case group**				**Control group**			
	N (%)	Mean ± SD	CI (95%)	P Value	N (%)	Mean ± SD	CI (95%)	P Value
**Gender**								
Male	38 (19.2)	19.32 ± 12.2	13–25.5	0.715^a^	88 (41.3)	2.5 ± 0.92	2.3–2.6	0.12^a^
Female	160 (80.8)	17.3 ± 11.82	14.3–20.2		125 (58.7)	2.6 ± 0.93	2.5–2.8	
**Marital statues**								
Married	165 (83.3)	18.62 ± 11.3	15.8–21.4	0.094^a^	175 (82.2)	2.53 ± 0.92	2.4–2.7	0.24^a^
Single	22 (11.1)	17.97 ± 14.8	6.7–29.3		29 (13.6)	2.65 ± 1	2.3–3	
Divorced	2 (1)	8.7 ± 9.71	5.4–20.8		2 (0.4)	3.15 ± 1.5	2.3–3.4	
Widow	9 (4.5)	9 ± 10.11	6.2–21.2		7 (3.3)	3.1 ± 0.4	2.7–3.4	
**Education**								
Primary	12 (6.1)	11.62 ± 5.4	4.5–20	0.452^a^	28 (13.1)	2.5 ± 0.8	2.18–2.81	0.442^a^
Elementary	82 (41.4)	20.2 ± 12.2	15.8–24.6		55 (25.8)	2.42 ± 0.86	2.2–2.7	
Diploma	58 (29.3)	14.8 ± 13.48	8.5–21		102 (47.9)	2.6 ± 0.92	2.45–2.8	
Graduate Diploma	15 (7.6)	15.9 ± 3.9	12.8–18.9		16 (7.5)	2.7 ± 1.2	2–3.5	
B.Sc.	27 (13.6)	19 ± 13	10.5–27.8		12 (5.6)	2.7 ± 1.23	1.95–3.5	
M.Sc. and higher	4 (2)	18.8 ± 12.7	11–26.9		—	—	—	
**Occupation**								
Self-employment	17 (8.6)	14.7 ± 2.4	8.6–20.7	0.894^a^	46 (21.6)	2.4 ± 0.95	2.1–2.7	0.445^a^
Housewife	131 (66.2)	17.2 ± 12.4	13.7–20.7		110 (51.6)	2.6 ± 0.89	2.5–2.8	
University student	11 (5.6)	16 ± 4.9	9.9–22.1		8 (3.8)	2.8 ± 0.93	2–3.6	
Employee	23 (11.6)	20.8 ± 14.2	13.3–28.4		22 (10.3)	2.7 ± 1.14	2.1–3.2	
Unemployed	16 (8.1)	16.4 ± 1.9	14–18.7		27 (12.7)	2.46 ± 0.89	2.1–2.8	
**Age (year)**								
20–25	19 (9.6)	18.74 ± 11.1	10–27.2	0.465^a^	21 (9.9)	2.6 ± 0.96	2.2–3	0.253^a^
26–35	50 (25.3)	21.3 ± 13.3	15.5–27		56 (26.3)	2.7 ± 1	2.4–3	
36–45	53 (26.8)	17.6 ± 14.7	10.3–24.9		54 (25.4)	2.4 ± 0.77	2.2–2.62	
46–60	76 (38.4)	14.7 ± 8.1	11.7–17.8		82 (38.5)	2.6 ± 0.9	2.4–2.8	
**BMI**								
<18.5	12 (6.1)	21.7 ± 12.5	8.5–34.7	0.57^a^	10 (4.7)	2.8 ± 1.1	1.9–3.6	0.68^a^
18.5–24.9	52 (26.3)	16.7 ± 8.2	12.6–20.7		62 (29.1)	2.5 ± 0.88	2.3–2.7	
25–29.9	77 (38.9)	18.3 ± 13	13.5–23.1		91 (42.7)	2.6 ± 0.98	2.4–2.8	
30–39.9	53 (26.8)	16.7 ± 12.7	11.45–21.9		49 (23)	2.5 ± 0.86	2.3–2.8	
>40	4 (2)	18.9 ± 12.7	13–22.9		1 (0.5)	—	—	
**Fluoride Level**								
0–0.29 mg/L	59 (29.8)	11.85 ± 7	8.9–14.8	0.003^a^	65 (30.5)	2.2 ± 0.95	1.9–2.4	0.001^a^
0.3–0.5 mg/L	139 (70.2)	20.4 ± 12.65	16.9–23.8		148 (69.5)	2.75 ± 0.88	2.6–2.9	

^a^Kruskal-Wallis test.

**Table 4:** The results of multiple logistic regressions model for factors affecting the hypothyroidism in case and control groups, YGA (Yazd Greater Area), 2017.

Variable	Variable subgroups	(OR)*	(CI 95%)^**^	P Value
Gender	Male	1		0.0001
	Female	2.5	1.6–3.9	
Family history of Thyroid Disease	No	1		0.0001
	Yes	2.7	1.6–4.6	
Amount of Water Consumption	One glass	1		0.001
	2–3 glass	1.73	0.5–5.9	0.382
	4–5 glass	4.1	1.2–14	0.024
	More than 5 glass	3.25	0.8–11.9	0.075
Exercise	Yes	1		0.0001
	No	5.34	3.2–9	0.0001
	Sometimes	3.66	2–6.6	0.001
Diabetes	No	1		0.001
	Yes	3.68	1.7–8	
Hypertension	No	1		0.013
	Yes	3.22	1.3–8.2	
Drinking Water Fluoride	0–0.29 mg/L	1		0.86
	0.3–0.5 mg/L	1.034	0.7–1.53	

*Logistic Model (Enter), Adjusted odds ratio (OR), **Confidence intervals (CI 95%).

As shown in Table 4, the variables that had greater effects on thyroid diseases remained in the model, such as gender, family history of thyroid disease, amount of water consumption, physical activity, as well as diseases such as type 2 diabetes and hypertension. The adjusted OR of hypothyroidism was 2.5 (CI 95%: 1.6–3.9) times greater in females, which is in agreement with another study^31^. The adjusted OR of hypothyroidism for those with a family history of hypothyroidism was 2.7 times (CI 95%: 1.6–4.6) higher than others. The adjusted OR of hypothyroidism was 5.34 (CI 95%: 3.2–9) times greater for those who were physically inactive as compared to their active counterparts. The adjusted OR of hypothyroidism for diabetic patients was 3.7 (CI 95%: 1.7–8) times higher than in healthy people. The adjusted OR of hypothyroidism for hypertensives was 3.2 (CI 95%: 1.3–8.2) times more than others. Individual fluoride intake from drinking water obviously depends on the amount of water consumed as well as the fluoride concentration in the water. The results showed that those who consume larger amounts of water per day have an adjusted OR of 4.1 (CI 95%: 1.2–14).

This study was the first research based on the correlation between fluoride concentration in drinking water and thyroid hormones in Iran. A positive association was observed between the variables (P < 0.05). In this study, we obtained an unadjusted OR of about 1.034 (CI 95%: 0.7–1.53) for fluoride in drinking water when its concentration was less than the standard (0.2–0.5 mg/L). This finding is consistent with the Peckham study in England, which reported OR = 1.5 (CI 95%: 1.16–2) for hypothyroidism, where the maximum fluoride concentration was more than 0.7 mg/L. However, it is not clear due to the small difference in the concentration of fluoride, as can be seen from the correlation between fluoride in drinking water and the TSH hormone as shown in Table 2^32^.

## Conclusion and Recommendation

This paper compares measurements of the average amount of thyroid hormones (T3, T4, and TSH) in people with thyroid disease (specifically, hypothyroidism) and people without thyroid disease, with respect to fluoride concentrations in two levels 0–0.29 and 0.3–0.5 (mg/L) in drinking water and several other variables (gender, family history, water consumption, exercise, other disease conditions).

The major finding of this study is that TSH values are higher with a higher fluoride concentration in the drinking water, even for generally low fluoride concentrations. This is seen both in cases of untreated hypothyroidism and in controls. In multivariate regression logistic analysis, the independent variables associated with hypothyroidism were: gender (odds ratio: 2.5, CI 95%: 1.6–3.9), family history of thyroid disease (odds ratio: 2.7, CI 95%: 1.6–4.6), exercise (odds ratio: 5.34, CI 95%: 3.2–9), diabetes (odds ratio: 3.7, CI 95%: 1.7–8), hypertension (odds ratio: 3.2, CI 95%:1.3–8.2), amount of water consumed per day (odds ratio: 4, CI 95%: 1.2–14).

In other words, cases tend to have higher TSH values (greater impairment of thyroid function) with higher fluoride concentrations in the water. Controls, with normal thyroid function, also have higher TSH values with higher fluoride concentrations, even though their TSH values are still within the normal range. TSH values are higher (in both cases and controls) with higher levels of water consumption. This is consistent with an association between increased fluoride intake (due to increased water consumption) and increased TSH. It was found that F impacts human thyroid hormones, especially TSH and T3 even in the standard concentration of less than 0.5 mg/L.

Even after the addition of iodine to salt by the integrated program in Iran more than 27 years ago, this study showed that the problem remains unsolved. The results showed that those who consume larger amounts of water per day have an adjusted OR of 4.1 (1.2–14). Hence, the application of standard household water purification (such as reversed osmosis, electro dialysis, activated carbon filter, and other adsorption/ion-exchange methods) is recommended for patients with hypothyroidism since they have a higher consumption of drinking water. The purification systems can help remove fluoride that interferes with thyroid functions.

## Materials and Methods

This study was a case-control study, aimed at determining the correlation between thyroid hormones and fluoride levels in the drinking water in YGA. We ensured that all the methods were carried out in accordance with the relevant guidelines and regulations.

### Study area

Iran has four distinct climatic regions: moderate and humid, warm and dry, cold and mountainous, and warm and wet^33^. It has 31 provinces, and is located between 24°N and 40°N (latitude) and 44°E and 64°E (longitude)^34^. YGA is an area in the Yazd province of Iran. It is subdivided into four cities—namely Yazd, Zarch, Shahediyeh, and Hamidiya^34^. Yazd is one of the warm and dry cities of Iran with average rainfall of 60 mm (2.4 in) per year or 5 mm (0.2 in) per month. Its driest weather occurs in August, during the summer, with average rainfall of 0 mm (0 in), temperatures above 40 °C (104 °F) in blazing sunshine, and humidity less than 2%. Its mean ± SD of fluoride present in drinking water is 0.5 mg/L ± 0.27^8,27,35^. The main sources of drinking water are different wells with varying fluoride concentrations. This makes YGA an ideal area for this study.

### Sample size

The cases and controls were chosen from the Yazd Healthy Study (YaHS) project. YaHS is a prospective study that examines the health of people from YGA in 2014–2016. The total number of YaHS participants was 10,000, with people aged between 20 and 70 years, who were selected through the cluster sampling method. Details of Yazd Health Study has been published else where^36^. The cases consist of participants with thyroidal diseases, who were not yet being treated. The participants in the control group belonged to the YaHS project, and were aged 20–60 years. The participants in the control group did not suffer from any thyroid disease. The Peckham study was selected to calculate the sample size^32^. Out of the 8,724 YaHS participants, 693 people (8%) reported various thyroid diseases diagnosed by a doctor. From these, 198 cases and 213 controls were selected.

### Investigation stages

To collect data, a standard questionnaire was prepared containing 68 questions of parameters that were thought to impact the thyroid gland or were sources of fluoride intake. All the participants (cases and controls) were requested to answer the same. The parameters included: age, sex, education, BMI, condition of pregnancy, diet, job status, salary, family history of thyroid-related diseases, any other disease that may have led to thyroid operation (metabolism or autoimmune disease, cholesterol disease, diabetes, blood pressure disease, polycystic disease, liver and kidney disease, neurology disease, depression, hepatitis disease, cardiovascular disease), place of residence, exercise, smoking, alcohol and drug intake, daily, weekly, and monthly intake of fluoride, and fluoride intake from sources other than water—such as toothpaste, mouthwashes, and some foods that contain fluoride (tea, cabbage, broccoli, turnip, soya, peanut, spinach, type of consumed fish, amount of consumed fish, type of consumed salt). Each odds ratio (OR), confidence interval (CI 95%), and p-value were examined for these factors for both the case and control groups. The ones that had p-values less than 0.2 were used for the final logistic model. Participants (case and control groups) were asked to take the T3, T4, and TSH hormone tests in the Yazd central laboratory. The radio immunoassay (RIA) method was used to test T3, T4, and TSH hormone levels. Samples of the participants’ drinking water were analyzed to determine the concentration of fluoride in the water at the wastewater laboratory in the School of Public Health, University of Shahid Sadoughi of Yazd. Finally, a mixed logistic regression model was applied for the statistical analysis.

### Drinking water fluoride study

As the water resources of this region differ, the samples were selected from the living sites of the case and control groups. The amount of fluoride (mg/L) present in the drinking water was measured and its concentration was determined by the SPADANS method. The test number 8,029 was used from the Standard Methods for the Examination of Water and Wastewater^37^. In this colorimetric method, the reaction occurs between fluoride and a zirconium-dye lake. The resultants of the reaction between fluoride and zirconium-dye lake are a colorless complex anion (ZrF_6_^2−^) derived from fluoride, and the dye. When the amount of fluoride in drinking water increases, the color progressively becomes lighter^37^. We used a fluoride reagent solution with the following specifications: 500 mL (HACH), range: 0.02–2.00 mg/L F-. The color was analyzed with the help of the DR2000 spectrophotometer (by HACH, a German company) and a 580 nm wavelength.

### Statistical analysis data

The data collected from the experiments and questionnaires was analyzed using logistic regression models. Thereafter, Microsoft EXCEL 2013, IBM SPSS statistics 20, and Arc GIS 10 were applied for the data analysis.

### Compliance with Ethical Standards

This study is Compliance with Ethical Standards. this study funded by Environmental Science and Technology Research Center, Department of Environmental Health Engineering, Shahid Sadoughi University of Medical Sciences, Yazd, Iran.

### Ethical approval and informed consent

This article does not contain any studies with human participants performed by any of the authors. Case and control people choses with their consent and have been informed about study and they completed the questionnaire. Their thyroid hormones analyze in Yazd central laboratory.
